# Assessment of Caregiver’s Knowledge, Complementary Feeding Practices, and Adequacy of Nutrient Intake from Homemade Foods for Children of 6–23 Months in Food Insecure Woredas of Wolayita Zone, Ethiopia

**DOI:** 10.3389/fnut.2016.00032

**Published:** 2016-08-15

**Authors:** Motuma Adimasu Abeshu, Abdulaziz Adish, Gulelat D. Haki, Azeb Lelisa, Bekesho Geleta

**Affiliations:** ^1^John Snow, Inc (JSI)-Ethiopia, Addis Ababa, Ethiopia; ^2^Addis Ababa University, Addis Ababa, Ethiopia; ^3^Micronutrient Initiative-Africa, Addis Ababa, Ethiopia; ^4^University of Botswana, Gaborone, Botswana; ^5^Micronutrient Initiative-Ethiopia, Addis Ababa, Ethiopia; ^6^Ethiopian Public Health Institute, Addis Ababa, Ethiopia

**Keywords:** complementary feeding, children, nutrient density, homemade, estimated daily nutrient intake

## Abstract

Complementary feeding should fill the gap in energy and nutrients between estimated daily needs and amount obtained from breastfeeding from 6-month onward. However, homemade complementary foods are often reported for inadequacy in key nutrients despite reports of adequacy for energy and proteins. The aim of this study was to assess caregiver’s complementary feeding knowledge, feeding practices, and to evaluate adequacy daily intakes from homemade complementary foods for children of 6–23 months in food insecure woredas of Wolayita zone, Ethiopia. A cross-sectional study assessing mothers/caregiver’s knowledge and complementary feeding practice, adequacy of daily energy, and selected micronutrient intakes using weighed food record method. Multi-stage cluster sampling method was also used to select 68 households. Caregivers had good complementary feeding knowledge. Sixty (88.2%) children started complementary feeding at 6 months and 48 (70.6%) were fed three or more times per day. Daily energy intake, however, was significantly lower (*p* < 0.05) than estimated daily needs, with only 151.25, 253.77, and 364.76 (kcal/day) for 6–8, 9–11, and 12–23 months, respectively. Similarly, Ca and Zn intakes (milligrams per day) were below the daily requirements (*p* = 0.000), with value of 37.76, 0.96; 18.83, 1.21; 30.13, 1.96; for the 6–8, 9–11, and 12–23 months, respectively. Significant shortfall in daily intake of Fe (*p* = 0.000) was observed among the 6–8 and 9–11 months (3.25 and 4.17 mg/day, respectively), even accounting for high bioavailability. The complementary foods were energy dense. Daily energy, Ca, Zn, and Fe (except 12–23 months) intake, however, was lower than estimated daily requirements.

## Introduction

The transition from exclusive breastfeeding to family foods typically covers the period from 6 to 23 months of age. It is the time when malnutrition starts in many infants, contributing significantly to the high prevalence of malnutrition in children <5 years of age worldwide. During this time, complementary foods should be added to the diet of the child (1–3). It is needed to fill the gap in energy and iron and other essential nutrients, between what is provided by exclusive breastfeeding and the total nutritional requirements of the infant. This gap increases with age, demanding an increasing contribution of energy and nutrients especially iron, from foods other than breast milk (4). Therefore, it should be timely, adequate, and be given in a way that is appropriate for the age of the child, applying responsive feeding (5).

As the child ages, feeding practices must change in response to the child’s changing nutritional requirements, motor skills, and maturing digestive and immune systems (4, 6). However, poor feeding practices characterized by poorly timed introduction of complementary foods (too early or too late); infrequent feeding and; poor feeding methods, hygiene, and child care practices, take the highest tall on complementary feeding problems (7, 8).

Added to these is poor dietary quality of the foods served. The diets of children 6–23 months old are often characterized by too little variety; inappropriate consistency (food is too thin or too thick); too few essential vitamins and minerals, especially vitamin A, iron, zinc, and calcium; too few essential fatty acids; and too few calories among non-breastfed infants (9).

Animal-source foods are rich in protein, fat, and micronutrients, but their cost limits consumption (10, 11). Commercial, fortified food products are often beyond the reach of the poor. A growing proportion of people in developing countries do not have the physical access and economic capability to purchase fortified products (12).

Child malnutrition is unacceptably high in Ethiopia, characterized by both macro- and micronutrient deficiency and associated health problems (13). A combination of nutritionally inferior diets and improper feeding practices are major contributing factors for the development of childhood malnutrition (14). In Ethiopia, complementary foods are usually an extension of family foods (15, 16). Family foods provide insufficient amount of important nutrients (17), even when improved food recipes are used (18).

The complementary foods are prepared from locally available staples, such as cereals, legumes, and tubercles, and are based primarily or exclusively on plant-derived products (16, 19). Plant-based complementary foods usually have low mineral contents and high levels of anti-nutritional factors, such as phytic acid (also known as phytate) (20, 21). Unfortified complementary foods that are predominantly plant-based generally provide insufficient amounts of key nutrients often designated as “problem nutrients,” such as iron, zinc, and calcium, to meet the recommended nutrient intakes during the age range of 6–23 months (19, 22, 23). Starch-based complementary foods produce viscous porridges and gruels which are often known to be of low nutritive value and are characterized by low protein, low energy density, and high bulk (24).

Only handful literature is available regarding adequacy of nutrient intake from homemade complementary foods in Ethiopia. For instance, traditional corn-based porridge consumed as complementary foods in rural villages of Sidama zone of southern Ethiopia contains energy density of 53 kcal/100 g while kocho-based ones contained 49 kcal/100 g, both of which containing very low energy levels (25). In addition, mothers/caregivers usually dilute them to facilitate administration. However, this produces further reduction in the energy and nutrient density of the food (4, 19, 26). The recommended complementary feeding recipes for Ethiopian children 6–23 months old, published by FMOH (27), contains daily recipes which bases at least on one of the three alternative staple groups: maize/*enset, teff*/wheat/barley, and maize/sorghum.

In northern Wollo, Baye et al. (15) assessed nutrient intakes from complementary foods while the focus age groups were young children of 12–23 months. By this age, most children are developmentally ready and are able to consume family foods of solid consistency compared with younger age groups and, thus, escape challenges associated with consuming family foods (6). Similarly, Gibson et al. (19) assessed the energy and nutrient composition of plant-based complementary food (made from potato, kale, chickpea flour, oil, and water) used in Ethiopia and evaluated its adequacy for children of 9–11 months. Even if a single set of recipe is used, gaps in key micronutrient intakes have been reported. Other studies based on 24-h recall were also conducted, for instance, EDHS series. Such methods use dietary diversity scores (DDSs) to predict nutrient density and assume serving sizes to predict adequacy of complementary foods (28). Evidences show that 24-h recall method is subjected to measurement errors and recall biases (29). In addition, there are limited studies conducted that targets population groups with characteristics of chronic food insecurity.

For this study, therefore, a weighed food record method was used to assess the composition of homemade complementary foods following traditional methods and evaluated the adequacy of the actual amount consumed per day to satisfy nutritional requirements of infants and young children of age 6–23 months in selected food insecure woredas in Wolayita zone, Ethiopia. This study also evaluated caregiver’s complementary feeding knowledge status and what food items they use to prepare complementary foods at household level. It also assessed nutritional profile of the diets, with emphasis to energy, protein, and micronutrients – calcium, zinc, and iron. In addition, it evaluated whether the amount of energy and key nutrients (Ca, Zn, and Fe) the children consumed per day would be adequate to satisfy their estimated daily nutrient needs from complementary foods.

## Materials and Methods

### Study Area and Period

Adequacy of daily energy, protein, and selected micronutrients intake from homemade complementary foods for children of 6–23 months was evaluated in 2 of the 5 community-based nutrition (CBN) woredas in Wolayita zone. Dugna Fango and Kindo Koysha woredas are characterized by history of high rate of severe acute malnutrition, high poverty, and chronic food insecurity (30). Dugna Fango woreda has a population of 104,564, whereas Kindo Koysha has a population of 96,480 (31). The major food crops in these woredas include, in order of importance, maize, sweet potato, *enset* (false banana), *teff* (Eragrostistef), haricot bean, sorghum, Irish potato, yam, and cassava (31, 32).

The study was conducted from October 2014 to April 2015.

### Study Design

A cross-sectional population study using a weighed food record method was used to assess the nutrient composition and evaluate adequacy of amount consumed per day to satisfy the nutrient requirement of children of age 6–23 months from homemade complementary foods in selected food insecure woredas of Wolayita zone.

### Study Population

All children of age between 6 and 23 months living in Kindo Koysha and Dugna Fango woredas of Wolayita zone, who have been breastfed the first 6 months of life and history of breastfeeding at the time of the study.

### Samples and Sampling Design

#### Sample Size

The sample size was estimated after reviewing published articles that have employed weighed food record method as their study design. For instance, the sample size used in article published by Baye et al. (15) was 76 households, whereas Abebe et al. (25) used 58 households. Accordingly, for this study, a sample size of 68 households was used.

#### Sampling Design

From five CBN woredas in Wolayita zone, two woredas with characteristics of food insecurity and severe malnutrition (30) were selected using convenient sampling method. The Kebeles (lowest administrative unit) in each woreda were then stratified into urban, semi-urban, or rural. One representative kebele from the rural and semi-urban kebeles in each woreda was then selected randomly using lottery method. Urban kebeles were excluded from sampling frame as preliminary assessments indicated mixed use of commercial and homemade complementary foods for child feeding. Subsequently, the size of households sampled per kebele was based on Proportionate Sampling method as per their total population sizes. Individual households within the kebeles were finally picked from list of eligible ones compiled from health facility registry and health extension workers’ report using simple random sampling method.

### Sample Collection and Handling

#### Sample Collection Tools

Semi-structured questionnaire was customized from: “Guidelines for assessing nutrition-related Knowledge, Attitudes, and Practices: KAP Manual” (33), “Guidelines for measuring household and individual dietary diversity” (11), and “Indicators for assessing infants and young child feeding practices” (34), piloted, and further enriched based on feedback obtained before its use to collect data regarding homemade complementary foods. Knowledge of mothers/caregivers was assessed using partially categorized questions regarding complementary feeding. Daily food intakes were measured using high precision digital scales. Weighed samples of the diets were sampled for nutrient analysis.

#### Implementation of Tools

Four qualified and experienced nutritionists with knowledge of the study area were recruited and trained on the tool and data collection procedures for 2 days and dispatched for the data collection. One data collector per kebele and a supervisor per woreda were assigned to undertake the process.

#### Sample Collection Procedure

Mothers/caregivers were visited the day before to obtain their consent and asked to maintain regular feeding styles. One investigator per one to two households in the same neighborhood was assigned to cover the observation, measurement, and sampling of foods. Visits started early in the morning (07:00 EAT) and ended when the mother certifies that the child would not consume anything except breast milk until the next day (20:00 EAT).

The measurement involved identification of food types served on visit day, food items used as recipe, and measurement of the quantity of complementary food served and consumed by the child throughout the day. For dishes prepared from mixed recipes, foods items used for preparing the complementary food were identified and weighed separately before starting the mixing and cooking process. The final weight of the preparation was also measured later. From the food items included in the preparation, DDS was constructed.

Measurement to the nearest 0.1 g was made using SF-400 digital balances of 1–2000 g capacity. The scales were calibrated every morning, and same scales were used throughout. Individual servings were measured by weighing the plate and food both before and after the children were fed. Samples proportionate to individual servings were drawn throughout the day for further laboratory assays.

Other socio-demographic information, such as mother’s/caregiver’s age and level of education, household size, parity, and income, was collected by interviewing the mothers/caregivers using the data collection tool.

#### Food Sample Handling

The complementary foods sampled were transferred to sample collection bottles, tightly closed, labeled, and placed in temperature-conditioned cold boxes. At the end of each day, the samples were transferred to and maintained in refrigerators until they were drawn for laboratory assays.

#### Preparation of Laboratory Samples

The primary food samples collected from households were sorted and grouped by the age of the children (6–8, 9–11, and 12–23 months) into sample category 1, 2, and 3, respectively. Three composite samples were then prepared by crushing and mixing the primary samples to homogeneity using mortar and pestle. Laboratory samples weighing 200 g were randomly sampled from each category for further assays.

#### Procedures for Laboratory Analysis

Assay of nutrient composition of the diets was conducted using established analytical procedures (35). The Moisture content of the Complementary Foods was determined using AOAC Official Method 925.10, while AOAC Official Method 920.39 was used to analyze the crude fat content. AOAC Official Method 923.03, 962.09, 986.11, and modified AOAC 985.35 was used to analyze crude ash, crude fiber, phytic acid, and the micronutrients (Ca, Fe, and Zn) contents, respectively. The crude protein content was determined following procedures under ES ISO 1871:213. Carbohydrate content was determined using difference method as:

% Carbohydrate=100−(%Moisture+%Fat+%Protein+%Ash+%Crude Fiber)

The daily nutrient and energy intake was computed by multiplying observed nutrient levels and total foods consumed by the child per day.

### Data Analysis and Interpretation

Household information, dietary nutrient levels, and daily intake data were cleared, coded, and analyzed using SPSS Version 20. The findings were presented using tables, charts, and graphs, whereas proportion, mean, and SD were the tools used to describe the data.

Adequacy of daily energy, protein, and micronutrients (Ca, Fe, and Zn) intake in comparison to the estimated needs was then evaluated. Estimated daily needs from complementary foods for energy is 200, 300, and 550 (kcal/day); calcium is 336.0, 353.0, and 196.0 (mg/day); zinc is 4.2, 4.3, and 5.8 (mg/day), for the 6–8, 9–11, and 12–23 months, respectively. Similarly, adequacy of daily iron intake was evaluated assuming high, moderate, or low bioavailability for iron. Accordingly, estimated daily need (in milligrams per day) of 20.8, 20.8, and 11.8 for low; 10.8, 10.8, and 5.8 for moderate, and 6.8, 6.8, and 3.8 for high bioavailability among the 6–8, 9–11, and 12–23 months, respectively (22, 36).

For differences observed, statistical significance was tested at *p* < 0.05 using *t*-test.

### Ethical Consideration

Ethical clearance from Addis Ababa University, Southern Nation Nationalities and Peoples Regional Health Bureau, Wolayita Zonal Health Department and each woredas was secured before commencing the study. At household level, informed oral consent was obtained from mothers/caregivers to proceed the data collection process. Utmost care has been taken to maintain the confidentiality of the participants during analysis and dissemination of findings.

## Results and Discussion

### Background Characteristics of the Respondents

The average household size was 5.2 (persons per household). The age distribution of the population surveyed shows 98.8% of the total populations were under the age of 49 years. Children between 6 and 23 months of age, however, constituted only 19.89% of the total population surveyed. The great majority of the population were between 15 and 49 years (42.90%) followed by the 5 and 14 years age group (28.41%). Almost all of the respondents (98%) were of Wolayita ethnicity and were followers of Protestant religion (60.8%), followed by Orthodox (38.1%) and Muslim (1.1%). Table 1 below provides summary of selected socio-demographic characteristics of the households surveyed.

**Table 1:** **Background characteristics of sampled households in Dugna Fango and Kindo Koysha woredas, Wolayita zone**.

Background variable		Woreda										Total	
		Dugna Fango				Woreda (%)	Kindo Koysha				Woreda (%)		
		Dugna Sore		Kerchecha			Sorto		Fechena				

		*N*	%	*N*	%	%	*N*	%	*N*	%	%	*N*	%
Gender	Male	36	56.25	43	51.81	53.74	65	45.45	32	51.61	47.32	176	50.00
	Female	28	43.75	40	48.19	46.26	78	54.55	30	48.39	52.68	176	50.00
	Total	64		83	100.00	100.00	143	100.00	62	100.00	100.00	352	100.00
Age ranges	<6 months old	0	0.00	0	0.00	0.00	1	0.70	0	0.00	0.49	1	0.28
	6–23 months old	12	18.75	17	20.48	19.73	29	20.28	12	19.35	20.00	70	19.89
	24–59 months old	6	9.38	4	4.82	6.80	15	10.49	0	0.00	7.32	25	7.10
	5–14 years	17	26.56	27	32.53	29.93	35	24.48	21	33.87	27.32	100	28.41
	15–49 years	26	40.63	35	42.17	41.50	62	43.36	28	45.16	43.90	151	42.90
	50–64 years	3	4.69	0	0.00	2.04	1	0.70	1	1.61	0.98	5	1.42
	>65 years	0	0.00	0	0.00	0.00	0	0.00	0	0.00	0.00	0	0.00
	Total	64	100.00	83	100.00	100.00	143	100.00	62	100.00	100.00	352	100.00
Educational status	Illiterate	35	54.69	40	48.19	51.02	82	57.34	28	45.16	53.66	185	52.56
	Read and write	2	3.13	1	1.20	2.04	3	2.10	1	1.61	1.95	7	1.99
	Elemen. (1–8)	20	31.25	29	34.94	33.33	40	27.97	26	41.94	32.20	115	32.67
	HighSch. (9–12)	6	9.38	12	14.46	12.24	17	11.89	7	11.29	11.71	42	11.93
	College/Univ.	1	1.56	1	1.20	1.36	1	0.70	0	0.00	0.49	3	0.85
	Total	64	100.00	83	100.00	100.00	143	100.00	62	100.00	100.00	352	100.00

**Figure 1:**
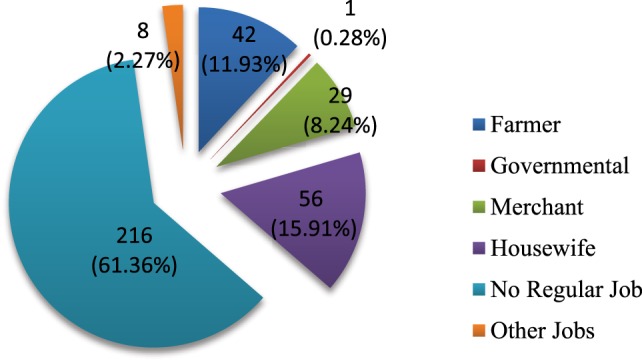
**Occupational status of household members**.

Literacy rate was very low (Table 1). Less than half (47.4%) of the total population were literate enough and capable of reading and writing. Out of these, 32.7% of them have attended elementary schools (1–8 grade), 11.9% have reached high school (9–12 grade), and only 0.9% have received some form of college training. Among the households members, majority (61.4%) of them have no formal jobs, whereas 11.9% of them were farmers (mostly the heads of the households) and 8.2% of them were merchants (Figure 1).

**Figure 2:**
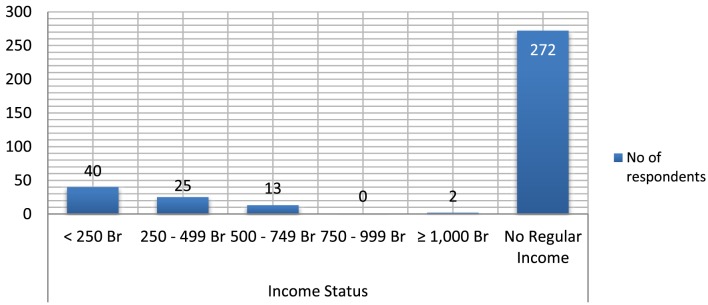
**Income distribution of respondents and household members**.

Great majority (272, 77.27%) of the household members surveyed have no regular source of income. The respondent’s feedback regarding their average monthly income (in ETB) shows that 40 (11.36%) of them had monthly earning of <250, while 25 (7.1%) and 13 (3.69%) of them had monthly earning of 250–499 and 500–749 ETB per month, respectively (see Figure 2 below).

Sixty-eight children/households were included in the study. Out of these, 28 (42.2%) households were selected from Dugna Fango Woreda, while the remaining 40 (58.8%) were from Kindo Koysha Woreda. Twelve (17.6%) households were selected from Dugna Sore and 16 (23.5%) were from Kercheche kebele of Dugna Fango. In addition, 29 (42.6%) of the households were from Sorto, and the remaining 11 (16.2%) households were selected from Fechena kebele of Kindo Koysha Woreda.

**Figure 3:**
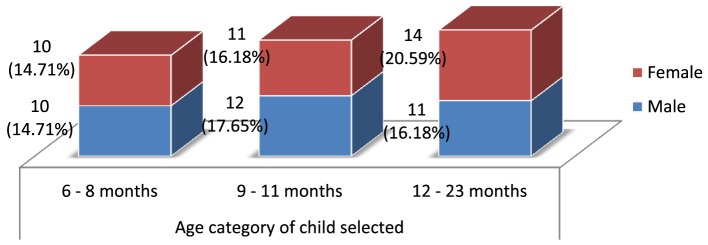
**Age distribution and gender composition of the children selected for the study**.

The age distribution was devised in such a way that sufficient number of children be included into one of the following three categories: children between 6 and 8 months, 9 and 11 months, and 12 and 23 months age groups (Figure 3). Out of the total sampled, 20 (29.41%) of them were between 6 and 8 months, while 23 (33.82%) and 25 (36.76%) were between 9 and 11 months and 12 and 23 months, respectively. Equal appropriation was given for children from both genders during the selection of households. Out of the 68 children selected for the study, 33 (48.53%) of them were males, and the remaining 35 (51.47%) were females.

### Knowledge of Complementary Feeding among Mothers/Caregivers

History of breastfeeding was used as one of the parameters for inclusion into the study. Therefore, all children included in the assessment were breastfed and breastfeeding at the time of data collection. According to WHO recommendation, complementary feeding should start by the time a child reaches 6 months and continue until 23 months and beyond (8). Review of the age of introduction of complementary foods shows almost all (88.2%) of the children investigated started complementary feeding by the age of 6 months, while the earliest age for complementary food introduction was at 4 months (2.9%). In addition, 98.5% of the respondents have continued until and beyond the age of 24 months.

The practice of starting complementary feeding at the recommended age was better when compared with the national figure. According to DHS (13), only 49.0% of children started complementary feeding at the age of 6 months. Foods other than breast milk were first introduced at as early age as <1 month. Even when compared with the national target to increase the percentage of 6- to 8-month-old infants on complementary food and continued breastfeeding from 54 to 65% over 2011 to 2015 periods (37), the level of complementary feeding practice observed surpasses the national target.

The difference between findings of this study and that of the national figure, in part, could be attributed to improved knowledge regarding infant and young child nutrition. Majority of respondents were knowledgeable about complementary feeding, on aspects of when to start (97.1%), benefits of complementary feeding (92.6%), how to prepare (85.3%) and keep hygiene (75.0%) of the complementary foods, as well as frequency of complementary feeding (Table 2). Health Extension Workers played paramount role by serving as the prime source of complementary feeding information for 98.5% of them.

**Table 2:** **Knowledge and practice of respondents on selected complementary feeding parameters**.

Complementary feeding (CF) knowledge and practice parameters		Respondents response against recommendations	
		No	% of total resp.
How long after birth to start breastfeeding	<1 h^a^	36	52.9
	1–6 h	30	44.1
	After 6 h	2	3.0
Age of introduction of CFs	4–6 months	3	4.4
	At 6 months^a^	60	88.2
	After 6 months	5	7.4
Knowledge about CF	When to start CF^a^	66	97.1
	Benefit of CF^a^	63	92.6
	How to keep hygiene^a^	51	75.0
	How to prepare CFs^a^	58	85.3
	Frequency of feeding^a^	46	67.6
	Others	4	5.9
Continued breastfeeding	Until and beyond 24 months^a^	67	98.5
	During illness^a^	67	98.5
	After recovery^a^	68	100.0
Source of CF information	Health extension workers	67	98.5
	Radio/TV	1	1.5
	Health volunteers	11	16.2
	Neighbors	3	4.4
	Others	6	8.8

*^a^Desired complementary feeding knowledge and/or practices (7)*.

### Preparation and Nutrient Composition of Homemade Complementary Foods

Caregivers should prepare appropriate complementary food reasonable number of times per day. Literatures suggest that the frequency should be in such a way that it minimizes holding over prepared food from one meal to the next and the risk of microbial contamination and optimizes the time and effort required by caregivers to prepare such foods (7). Among the subjects observed in this study, the caregivers cook complementary foods an average of 2.26 times per day (range of 1–4), which is online with the recommended average levels.

Children are often fed multiple foods in a given day. A look at the number of food types used for feeding shows that a child is offered an average of 2.79 food types in a day. Along the spectrum of households, the number of food types used ranged from 1 to 6/day. Twenty-one (30.9%) households served three food types, 20 (29.4%) served two, and 9 (13.2%) households served only one food type. The remaining 18 (26.5%) households have served four or more food types to their child per day.

Laboratory assay of the sampled complementary foods was conducted to identify their nutrient compositions. From the macronutrients, assay of protein, fat, and carbohydrate composition was made. From the micronutrients, nutrient analysis was conducted for calcium, iron, zinc, and ash content, in general. In addition, the moisture, crude fiber, and phytic acid content were analyzed. Results of the assay are presented in Table 3.

**Table 3:** **Energy, nutrient, and anti-nutrient content (per 100 g as eaten/edible portion) of homemade complementary food**.

Nutrient profiles	Amount per 100 g (EP) by age category of children		
	6–8 months	9–11 months	12–23 months
Energy (kcal)^a^	91.28	124.24	141.16
Fat/lipid (g)	2.80	3.92	2.64
Protein (g)	3.11	2.66	3.50
CHO (g)^b^	13.41	19.58	25.85
Moisture (%)	75.63	69.59	64.07
Calcium (mg)	22.79	9.22	11.66
Iron (mg)	1.96	2.04	2.87
Zinc (mg)	0.58	0.59	0.76
Ash (g)	4.41	4.41	3.18
Fiber (g)	0.63	0.76	0.76
Phytate (mg)	23.31	59.31	10.50

*^a^The energy content was calculated using conversion factor for respective macronutrients*.

*^b^Carbohydrates content was calculated using % difference method*.

*CHO, carbohydrates*.

Energy density was calculated from the energy yielding nutrients using their respective conversion factors. Accordingly, the energy density per 100 g of complementary foods served to children of age between 6 and 8 months was found to be 92.28 kcal, while 124.24 and 141.16 kcal of energy were found per 100 g of foods served to the 9–11 and 12–23 months of age, respectively. This result shows that energy density increased with the age of the children.

### Feeding Practice: Size, Frequency, and Impact of Consistency on Amount Consumed

Complementary food should fill gaps in nutritional requirement from breastfeeding alone, starting from age of 6 months. In order for the infant and young child to get sufficient amounts of nutrients, multiple feeding per day is recommended. For an average healthy breastfed infant, complementary foods should be provided 2–3 times per day at 6–8 months, and 3–4 times per day at 9–11 and 12–24 months of age, with additional nutritious snacks (such as a piece of fruit or bread or chapatti with nut paste) offered 1–2 times per day, as desired (7, 8). For this study, a serving was qualified as a serving episode when the amount/weight of the food is >10 g.

In line with these recommendations, the average number of serving episodes observed per day during this study was 3.0, with a slight difference among the different age groups (Table 4). The same level of meal frequency was reported in study conducted by Baye et al. (15). However, this does not guarantee intake of adequate nutrient from complementary foods. Rather, adequate intake is ensured only when sufficient amount of diet with good nutritional quality is consumed.

**Table 4:** **Number of serving episodes per day by age of the children**.

		Serving/day (average)		Dispersion		Number of serving episodes per day		
				SD	Range	2	3	4
Age Cat.	6–8 months	2.90	0.553	2	4 (20.0%)	14 (70.0%)	2 (10.0%)
	9–11 months	2.91	0.515	2	4 (17.4%)	17 (73.9%)	2 (8.7%)
	12–23 months	3.16	0.54	2	2 (8.0%)	17 (68.0%)	6 (24.0%)
	6–23 months	3.00			10 (14.7%)	48 (70.6%)	10 (14.7%)

In order to quantify the actual amount of foods served to a given child per day, all servings were weighed and/or measured throughout the day. Results from the measurement show the amount of food served to a child per day varied from 57.00 to 909.00 g, with an average amount of 307.57 g (SD = 165.57 g). The amount served to a child in a day also varied among the different age groups. On average, for instance, children of age between 6 and 8 months were served 263.30 g (SD = 109.52 g) per day compared with 317.09 g (SD = 161.78 g) for 9–11 months and 334.92 g (SD = 201.35 g) for the 12- to 23-month age categories.

The amount of complementary food consumed was very low. Measurement of the amount of food actually consumed by a child per day among all age groups shows an average of 212.82 g (183.47, 271.50). The minimum amount of food consumed per day was found to be 47.00 g, whereas a maximum weighed consumption of 658.00 g was also observed.

**Figure 4:**
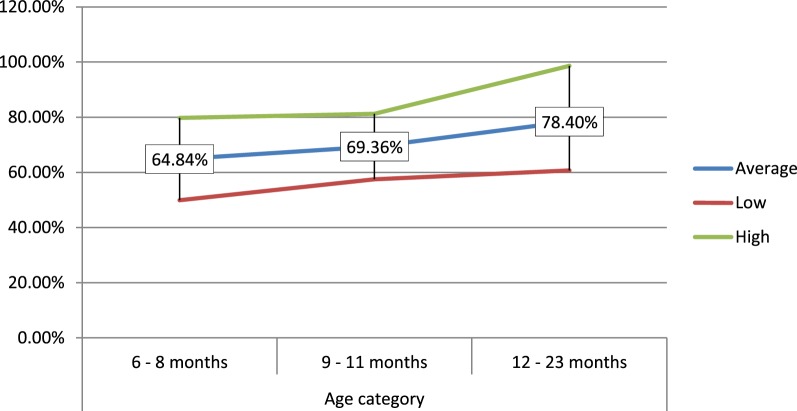
**Portion of daily servings (in %) consumed by age of the children**.

In percentage values, the 6–8 months consumed 64.84% of the amount of food served to them, which is lower in comparison to the 9- to 11-month (69.36%) and 12- to 23-month age groups (78.40%) (Figure 4).

The amount of food consumed per day increased as the age of the children progressed. Even so, the children’s functional gastric capacity, i.e., the amount of food consumed at single meals, was significantly lower than estimated level for their age. The gastric capacity per kilogram of body weight was found to be 19.96 (15.37, 24.55) g/kg for 6–8 months, 21.50 (17.83, 25.17) g/kg for 9–11 months, and 22.47 (17.41, 27.53) g/kg for 12–23 months (Table 5). Even if this is higher than the amount reported by Baye et al. (15) for children of 12–23 months, which is 9.00 g/kg, it is much lower than the theoretical gastric capacity of 30 g/kg body weight per meal (7, 22). This may be due to lack of responsive feeding, inappropriate consistency of the foods, or due to underlying health conditions.

**Table 5:** **Comparison of selected complementary feeding practice indicators by the age to recommended levels**.

	Complementary feeding practice mean score by age and recommended levels					
	6–8 months		9–11 months		12–23 months	
	Observed	Recommended	Observed	Recommended	Observed	Recommended
Gastric capacity/kg (g/kg), and C.I.^b^	19.96^a^ (15.37, 24.55)	30.0	21.50^a^ (17.83, 25.17)	30.0	22.47^a^ (17.41, 27.53)	30.0
Functional gastric capacity (g/meal or day), and C.I.^b^	165.70^a^ (127.54, 203.86)	249.00	204.26^a^ (169.35, 239.17)	285.00	258.40^a^ (200.18, 325.10)	345.00

*^a^Observed values lower than recommended levels based WHO recommendations (7, 8)*.

*^b^Functional gastric capacity is calculated based on median body weight at 7 months for 6–8, 10 months for 9–11, and 18 months for 12–23 ages, which is 8.3, 9.5, and 11.5 kg, respectively (22)*.

*C.I. is an acronym for Confidence Interval of the mean values*.

The consistency of a food affects child feeding as the child may be unable to consume more than trivial or may take so long to eat that food. In this study, four food consistencies: solid, semi-solids (thick like porridges), semi-solid (thin like soup), and liquids were observed. Solid preparations were predominantly served and was observed among 51 (75.0%) of the households’ daily food servings. This is followed by thick semi-solid preparations (28, 41.2%), liquid (21, 30.9%), and thin semi-solid preparations (17, 25.0%). Variation in food consistencies by age was also observed.

The consistency of complementary foods needs to be appropriate for the child’s stage of neuromuscular development. The use of solid foods in the diets of the children was rampant. Three-fourth (75.00%) of children of 6–23 months were served solid foods. Evidences suggest that by 12 months, most infants are able to consume “family foods” of a solid consistency. Until such time, the infants develop the ability to chew and swallow foods, semi-solid or pureed foods are recommended (6, 22, 38). In reality, this study shows that more than half (55.00%) of children between 6 and 8 months were served solid foods, whereas about one-third (32.71%) of amount served remained unconsumed. In line with these developmental changes, variation in quantity of food consumed per day was observed as the consistency of the diet changes (Table 6).

**Table 6:** **Consistency of the foods served and portion of servings consumed by the child (in %) from the 4 food consistencies**.

		% households who have served these food consistencies				Portion of servings consumed by the child (in %) from the four food consistencies			
		Solid	Thick semi-solid	Thin semi-solid	Liquid	Solid	Thick semi-solid	Thin semi-solid	Liquid
Age category	6–8 months old	55.00	55.00	40.00	40.00	67.29	68.58	70.45	59.72
	9–11 months old	69.60	57.00	22.00	30.40	71.10	63.83	73.07	75.721
	12–23 months old	96.00	16.00	16.00	24.00	79.61	57.12	67.94	70.392
	6–23 months old	75.00	41.20	41.20	30.90	74.28	64.74	70.632	68.10
Mean intake (%)						74.28	64.74	70.63	68.10

### Adequacy of Energy and Protein Intakes from Complementary Foods

The energy needs from complementary foods increases as the age of child increases. The total energy requirement estimated for healthy breastfed infants is approximately 615 kcal/day at 6–8 months, 686 kcal/day at 9–11 months, and 894 kcal/day from 12 to 23 months (39). The energy needs from complementary foods for infants with average breast milk intake in developing countries are approximately 200 kcal per day (29%) at 6–8 months of age, 300 kcal per day (55%) at 9–11 months of age, and 550 kcal per day (71%) at 12–23 months of age (7, 38). Complementary foods are expected to fill these deficits in energy between daily requirement and the amount obtained from breastfeeding.

In reality, the amount of energy consumed per day, in all age groups, was found to be well below the minimum recommendations (*p* < 0.05). This finding is in line with other findings. Baye et al. (15) also reported inadequacy of energy consumed per day among children of age 12–23 months in North Wollo.

**Figure 5:**
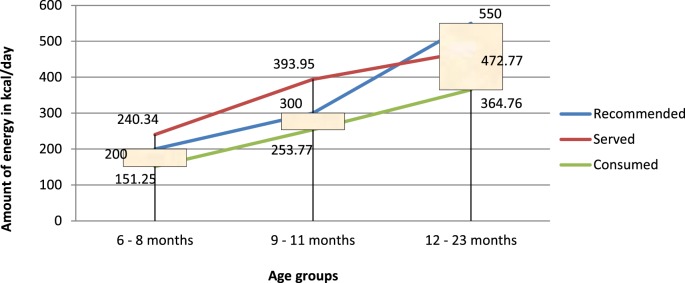
**Comparison of energy served and/or consumed (in kilocalories per day) to recommended intakes**.

One important finding, perhaps a paradox, is that the total amount of energy in the diets served for all, except the 12- to 23-month age groups, was more than the recommended levels. Even if adequate quantity/amount of diet containing good energy density were served, the amount of energy consumed per day fell short of daily age-specific recommendation for all age groups (Figure 5).

This mismatch, perhaps, may also be a reminder on the need for caution on projecting adequacy of amounts consumed based only on the energy density, by taking assumptions on the meal frequency and amount of food could have been consumed during the day. For instance, study conducted by Gibson et al. (19) concludes adequate consumption of energy by children of age 9–11 months assuming that: the Infants received three feedings of each complementary food per day as well as breast milk; and the amount of food consumed per feeding was 250 g/250 ml.

**Figure 6:**
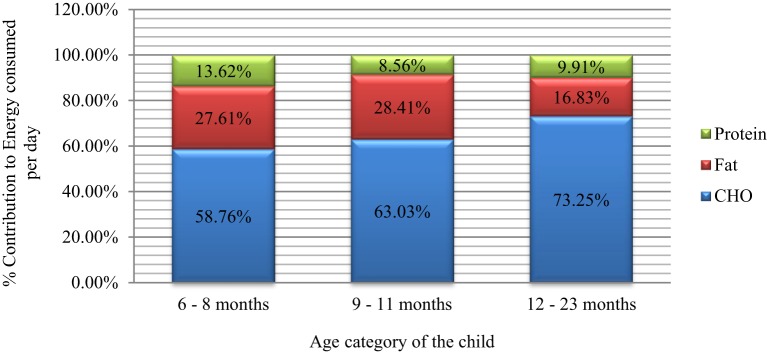
**The % contribution of macronutrients to energy consumed/day by age of the children**.

The energy yielding macronutrients contributed to the total daily energy consumption to varying extents. Carbohydrates were the primary and major contributors (>55%) to the daily energy consumed with increasing trend by age (Figure 6). Carbohydrates contributed to 58.76% (88.88 kcal out of 151.25 kcal), 63.03% (159.96 out of 253.77 kcal), and 73.25% (267.2 out of 364.76 kcal) of energy for the 6–8, 9–11, and 12–23 months, respectively.

The optimal amount of fat in the diets of infants and young children ranges from 30 to 45%. The amount of fat needed from complementary foods to satisfy daily requirement for infants and young children depends on the level of breast milk intake. For those with low breast milk intake, complementary foods should provide dietary fats appropriating to 34, 38, and 42% of daily energy requirements for 6–8, 9–11, and 12–23 months, respectively. With adequate breast milk intake, however, the requirement from complementary foods is 0 g/day (0%) at 6–8 months, 3 g/day (5–8%) at 9–11 months, and 9–13 g/day (15–20%) at 12–23 months (7).

The protein requirement of infants and young children increases with their age. The amount of protein (in grams per day) required to satisfy their daily nutritional requirement is 9.1 g for 6–8 months, 9.6 g for 9–11 months, and 10.9 g for 12–23 months. Breast milk provides a significant portion of daily protein requirement of infants and young children. Assuming average breast milk intake, the amount of protein needed from complementary foods is 1.9 g/day at 6–8 months (21%), 4.0 g/day at 9–11 months (42%), and 6.2 g/day (57%) at 12–23 months (7, 40).

The ratio of energy from protein to total energy contained in the complementary foods (protein–energy ratio) was 13.6% for 6–8 months, 8.5% for 9–11 months, and 9.9% for 12–23 months, all of which were significantly higher than the recommendations (Table 7).

**Table 7:** **Comparison of protein density (g/100 kcal) and Protein:Energy ratio to the recommendations, by age of the children**.

Age category (months)	Protein:energy (PE) ratio	
	In foods consumed (%)	Minimum recommendation (%)^a^
6–8	13.62*	5.1–6.3
9–11	8.50*	4.8–6.0
12–23	9.90*	4.3–5.7

*^a^Value adopted from Ref. (41)*.

*Mean for Observed is significantly higher than the Recommended at *p* < 0.05 (*p* = 0.000).

With this regard, the amount of proteins and fats consumed per day was adequate. Similar findings were reported for protein density in homemade complementary foods prepared in other parts of Ethiopia (15, 19).

### Adequacy of Calcium, Iron, and Zinc Intakes from Complementary Foods

Breast milk is relatively low in several other micronutrients, even after accounting for bioavailability. The percentage of total daily requirement for micronutrients needed from complementary foods, therefore, ranges from 30 to 97% (39). Thus, complementary foods must provide relatively large proportions of micronutrients, such as iron, zinc, phosphorus, magnesium, calcium, and vitamin B6 (7, 23, 39). Given the relatively small amounts of complementary foods that are consumed at 6–23 months, the nutrient density of complementary foods therefore needs to be very high (7).

Micronutrients, such as calcium, iron, and zinc, continue to be problem nutrients. When compared with the amount needed to satisfy their nutritional needs, none of the children from all age groups (6–8, 9–11, and 12–23 months) obtained sufficient amount of calcium, iron, or zinc (milligrams) per day. The only exception was observed on iron consumption in the 12- to 23-month age groups. Children belonging to this age group obtained sufficient amount of iron per day when moderate-to-high bioavailability is assumed. Declining iron requirement (in milligrams per day) as the age of the children increased, not improved iron composition of the diets, is the reason for the differences observed (Table 8).

**Table 8:** **Micronutrients consumption per day compared with estimated daily nutrient needs from complementary foods by age of the children**.

Nutrients		Age category of the children					
		6–8 months		9–11 months		12–23 months	
		Amount consumed	Recommended^a^	Amount consumed	Recommended^a^	Amount consumed	Recommended^a^
Ca (mg/day)		37.76	336.00*	18.83	353.00*	30.13	196.00*
Fe (mg/day)	Low bioavailability	3.25	20.80*	4.17	20.80*	7.42	11.80*
	Medium bioavailability		10.80*		10.80*		5.80**
	High bioavailability		6.80*		6.80*		3.80**
Zn (mg/day)		0.96	4.20*	1.21	4.30*	1.96	5.80*

*^a^Value for desired nutrient density adopted from WHO recommendations (22)*.

*Amount of micronutrients consumed per day is significantly lower than minimum daily requirement at α < 0.05 (p = 0.000).

**Amount of micronutrient consumed per day is higher than the minimum estimated daily requirement.

In many developing countries, commercial fortified food products are often beyond the reach of the poor. As a result, homemade complementary foods are frequently used during child feeding (12). The basic recipe food items used for the preparation of the complementary food commonly base on locally available staples while the choice of specific food item differs considerably between populations, owing to tradition, availability, and ease of access (40).

A commonly shared phenomenon about homemade complementary foods that are based on starchy roots and tubers or rice and available in many low-income countries is their frequent shortfall in amounts of selected essential micronutrients, such as calcium, iron, and zinc. By contrast, the recipes prepared from maize and legumes or other cereal mixtures and legumes had higher iron and zinc contents, but they also have considerably higher phytate contents (21). The net effect in both cases is that they would not meet the theoretical mineral requirements of young children due either to their low mineral content or as a result of low bioavailability, unless enriched with animal-source foods such. As a result, World Health Organization designates calcium, iron, and zinc as “problem nutrients” and deficiencies of these minerals can lead to adverse health consequences and restricted child growth and development (19, 36).

As these problem nutrients also are relatively low in breast milk, eminent deficiencies of these micronutrients inadvertently leads to adverse health consequences and restricted child growth and development (19, 21, 36).

## Conclusion

The homemade complementary foods were mostly an extension of family foods. Even if good knowledge on the basic complementary feeding practices among the mothers/caregivers was observed, the net daily intake of energy, Ca, Fe, and Zn by the children failed to fulfill the estimated daily nutrient requirements from complementary foods. Their functional gastric capacity was also very low.

### Recommendations

Additional studies would be required to substantiate the major findings reported in this assessment. Within the scope of this study, the age inappropriateness of some servings and high level of unconsumed leftovers would call for successive nutrition and health education to mothers/caregivers so as to institute responsive feedings. Targeted nutrition education would be required to enhance selection of nutrient dense food items, food diversification, adoption of processing methods that improve nutrient availability, and recommended ages for introducing/switching food consistencies. The gaps in nutrient intakes, especially the micronutrients, were very huge. Nutrition programs should therefore strengthen further and streamline micronutrient interventions so as to address such gaps and minimize the risk of associated malnutrition and ill-health on short- and long-term basis.

## Author Contributions

MA: conceived and designed the study, and performed all the data analyses. AA, GH, and AL: supervised the data collection and analyses. BG: wrote the first draft of the manuscript. All authors contributed to the interpretation of the data, revising the manuscript critically for important intellectual content, and approved the final version.

## Conflict of Interest Statement

The authors declare that the research was conducted in the absence of any commercial or financial relationships that could be construed as a potential conflict of interest.
